# Synthetic Applications of Intramolecular Thiol-Ene “Click” Reactions

**DOI:** 10.3390/molecules191119137

**Published:** 2014-11-19

**Authors:** Eoin M. Scanlan, Vincent Corcé, Aoife Malone

**Affiliations:** Trinity Biomedical Sciences Institute, Trinity College Dublin, 152-160 Pearse Street, Dublin 2, Ireland; E-Mails: vincent.corce@gmail.com (V.C.); maloneao@tcd.ie (A.M.)

**Keywords:** free radical, organic synthesis, carbohydrate, peptide

## Abstract

The intermolecular thiol-ene reaction is emerging as a highly efficient; free-radical mediated “click” process with diverse applications in biofunctionalisation and materials science. The related intramolecular thiol-ene reactions offer significant potential for the preparation of a wide range of sulphur containing heterocycles including synthetic therapeutics such as cyclic peptides and thiosugars. Herein, we review recent advances in intramolecular thiyl-radical mediated reactions and their applications for synthetic and medicinal chemistry.

## 1. Introduction

The ability to prepare structurally diverse heterocycles from acyclic starting materials using ring-closing reactions is one of the cornerstones of modern synthetic organic chemistry. Heterocycles are integral structural components of many natural products and therapeutics and developing efficient methodologies for their synthesis remains an area of intensive research focus for organic chemists. Free radical chemistry has provided a plethora of efficient methodologies for ring-closing reactions and many of these methodologies are routinely used to access complex heterocycles [1]. Whereas many of these methodologies incorporate cyclisation reactions involving carbon centered radicals, systems where the heteroatom is the reactive radical intermediate during the cyclisation step have also been investigated [2,3,4,5,6]. In this review article we will focus attention on one such ring-closing methodology, the intramolecular thiol-ene “click” reaction where a reactive thiyl radical undergoes an addition reaction onto an alkene to furnish a sulphur containing heterocycle. First described as a radical mediated process by Walling and co-workers in 1964 [7], this facile and high-yielding reaction has been applied to the synthesis of a diverse range of sulphur containing heterocycles. The review article will cover the synthetic scope of the methodology; discuss the factors affecting the regioselectivity of the cyclisation step for varying ring sizes and focus on recent literature examples of applications in organic synthesis.

## 2. Thiyl Radicals in Organic Chemistry

Thiyl radicals are highly versatile reactive intermediates that are known to undergo a wide range of addition reactions to unsaturated systems including alkenes, alkynes, thiocarbonyl and isonitrile groups [8]. Thiyl radicals are readily generated via the interaction of a sulphur containing molecule with a radical species, for example a thiol in the presence of an alkyl radical [9]. The S-H bond dissociation energy for alkyl thiols is generally accepted to be around 87 kcal·mol^−1^ regardless of the overall structure of the alkyl residue [10] and this facilitates the use of common initiators such as azo-compounds or peroxides that generate alkyl or alkoxyl radicals for thiol-mediated radical transformations. Thiyl radicals may also be efficiently generated on treatment of a thiol with a single-electron oxidant such as a Mn(III) compound [11] or through direct photolysis of the S-H bond under UV irradiation. All of these various initiation modes have been applied to the intramolecular process. Thiyl radicals play a vital role in nature in a broad range of biochemical transformations, functioning with a high yield and exquisite specificity. The best known example is the participation of a protein based thiyl radical in the deoxygenation of ribonucleotides, a transformation that is used by all living organisms in the synthesis of DNA [12,13]. Due to the mild reaction conditions, high yields and the ability to function in a fully or partially aqueous environment, it is therefore not surprising that thiyl radicals have been extensively utilised by organic chemists as reactive intermediates for a range of radical mediated transformations [8].

## 3. The Intermolecular Thiol-Ene Reaction

The reversible addition reaction between a thiyl radical and an alkene is commonly known as the thiol-ene reaction and it gives rise to a robust thioether linkage [14]. The intermolecular reaction is the most widely used synthetic application of thiyl radicals and has been employed for a range of applications including polymerisation reactions [9], biofunctionalisation [15,16] and ligation reactions [17]. The intermolecular thiol-ene reaction has been extensively reviewed elsewhere previously [8,9] and will not be discussed in detail here.

## 4. The Intramolecular Thiol-Ene Reaction

The intramolecular thiol-ene reaction is mechanistically related to the intermolecular process, but whereas for the intermolecular process a high degree of regioselectivity is usually observed, for the intramolecular process the regioselectivity of the radical addition reaction is much more difficult to predict and control, in particular for cyclisation reactions onto unsubstituted terminal alkenes. The regioselectivity of the addition of thiyl radicals to olefins for the intermolecular process can be rationalized in terms of the stability of the intermediate carbon-centered radical formed upon addition of the thiyl radical to the less substituted olefinic carbon atom [18]. The related cyclisation reactions of alkoxyl and aminyl radicals are closely matched to those of carbon centered radicals but due to the larger bond length of C-S^●^ bond compared to C-C^●^, C-O^●^ or C-N^●^, the orthogonal overlap of the thiyl radical with the double bond allows for cyclisation to occur at either position of the alkene [19]. A molecular orbital explanation for the absence of regioselectivity in the intramolecular thiol-ene reaction has been proposed by Baldwin. The rationale for the allowed cyclisation reaction at the terminal position is due to the presence of the unoccupied 3d orbitals on the sulphur atom that undergo back bonding interactions with the filled π-orbital of the double bond thereby reducing the geometric constraint usually associated with terminal ring closing reactions [20]. The ability of the thiol-ene reaction to proceed via both *exo* and *endo* cyclisation modes, coupled with the rapid reversible nature of the thiol-ene reaction makes it difficult to predict the outcome of these reactions and mixtures of isomers are often observed. However, careful control of the reaction conditions and choice of substrates can allow the reaction to be directed towards a single product and many examples of highly regioselective thiyl radical mediated cyclization reactions have been reported in the literature. The ability to access products of varying ring size from a single starting material, combined with the potential access to various diastereoisomers, makes this a powerful tool for drug discovery and library synthesis. Competing intermolecular addition reactions are not a significant factor unless the reaction is carried out at a high concentration.

**Scheme 1:**
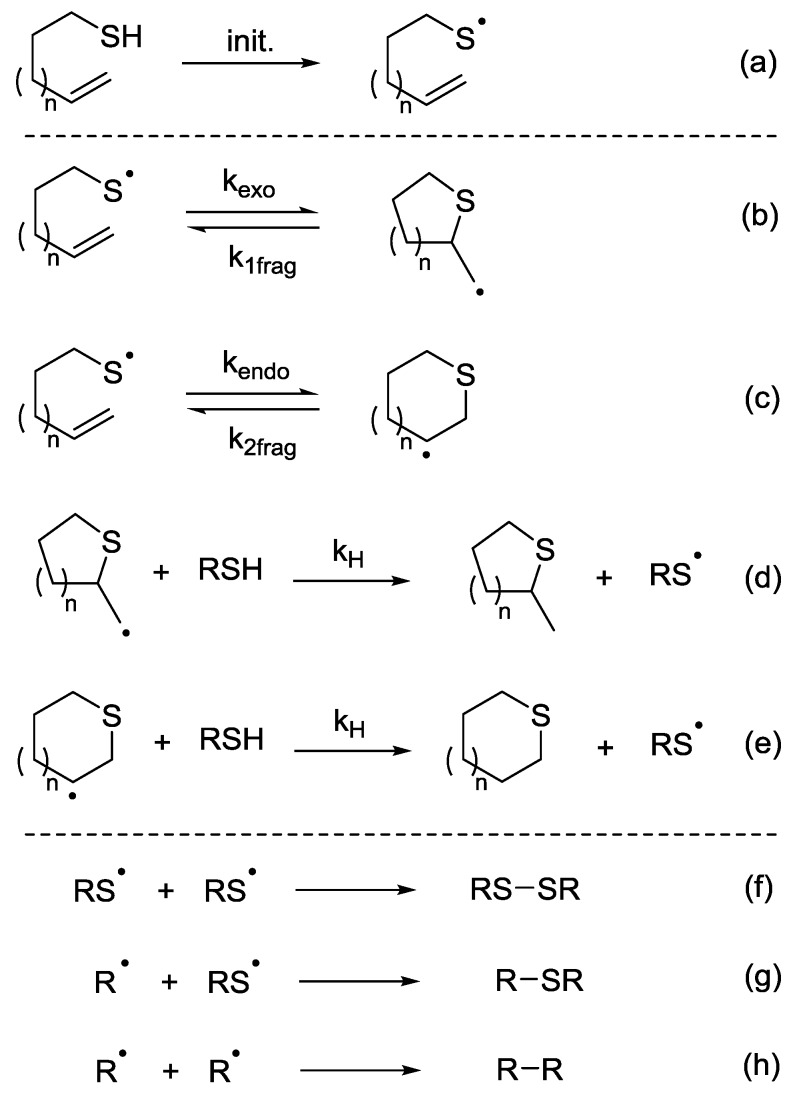
Initiation, Propagation and Termination steps for intramolecular thiol-ene reaction pathway.

The overall intramolecular thiol-ene process is outlined in Scheme 1. Following formation of the reactive thiyl radical (Scheme 1, Equation (a), initiation step), there are two potential pathways that the reaction can follow. The thiyl radical can cyclise in either an *exo* or an *endo* manner to furnish two possible ring sizes (Scheme 1, Equations (b) and (c)). In each case the cyclisation reaction is reversible so the stability of the radical that is formed upon cyclisation is crucial in determining the final product distribution. Reaction conditions are also important in directing the reaction towards the thermodynamic or kinetic products. Following the cyclisation step the resulting radical can then abstract a hydrogen atom from another molecule of thiol to give the heterocyclic thioether product and a sulfanyl radical which propagates the chain reaction (Scheme 1, Equations (d) and (e)). A number of termination steps can occur including disulfide formation, reaction of an alkyl radical with a sulfanyl radical and dimerization of two alkyl radical species (Scheme 1, Equations (f), (g) and (h)).

The distribution of products formed in the intramolecular thiol-ene process depends on a number of factors including the relative stability of the alkyl radicals formed after cyclization and the rate of the thiyl hydrogen atom abstraction step. The kinetics of the intermolecular thiol-ene polymerization reaction have been studied in detail by a number of groups and should translate well to the intramolecular process [17,18]. The most important factor governing the overall kinetics of the thiol-ene polymerization reaction is the ratio of the propagation rate (radical addition step) to the chain transfer rate (hydrogen transfer step). For the intramolecular process we may assume that the chain transfer step is rate limiting and that the overall process is first order with respect to thiol concentration. Due to the number of factors involved in determining the product distribution of the reaction, careful control of conditions and substrate design must be employed to achieve control over both regioselectivity and stereoselectivity.

## 5. Regioselectivity of Intramolecular Thiol-ene Ring-Closing Reactions

The following section discusses literature examples and general trends of intramolecular thiol-ene ring closing reactions for varying ring sizes.

### 5.1. 4-exo vs. 5-endo Systems

**Scheme 2:**
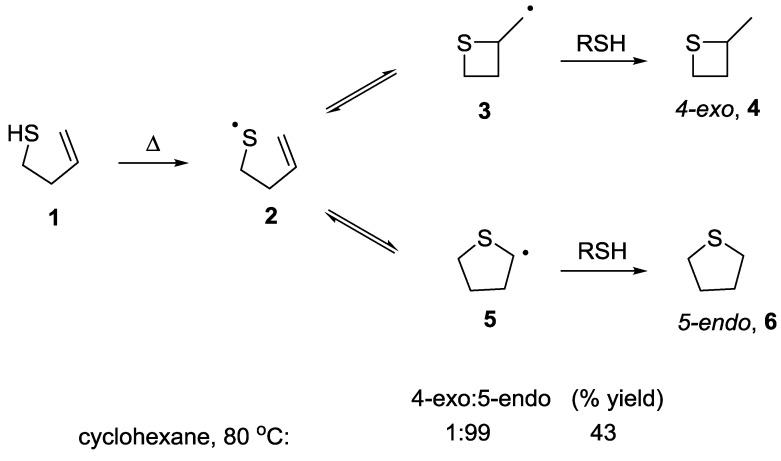
Results for 4-*exo vs*. 5-*endo* cyclisation reaction as reported by Surzur and co-workers.

There are very few literature examples where the 4-*exo vs*. 5-*endo* process for intramolecular thiol-ene cyclisation has been investigated. Surzur and co-workers reported that thermolysis of 3-butenethiol (**1)** in cyclohexane resulted in a 43% yield of the 5-*endo* product **6**, with <1% of the 4-*exo* product **4** observed (Scheme 2) [21]. ESR studies of the radical cyclisation confirmed the presence of the intermediate radical formed from the 5-*endo* cyclisation but none of primary alkyl radical resulting from the competing 4-*exo* cyclisation. Analogous results were reported on alkyl substituted 3-butenethiols and none of the 4-*exo* products were observed. Surzur proposed that the absence of the 4-*exo* product was due to an inefficient overlap of the thiyl radical with the *sp^2^* carbon required for the highly strained 4-*exo* cyclisation pathway. In addition, it was proposed that if the 4-*exo* product did form, the reverse ring opening reaction would take place extremely rapidly and therefore may not be trapped.

### 5.2. 5-exo vs. 6-endo Systems

**Scheme 3:**
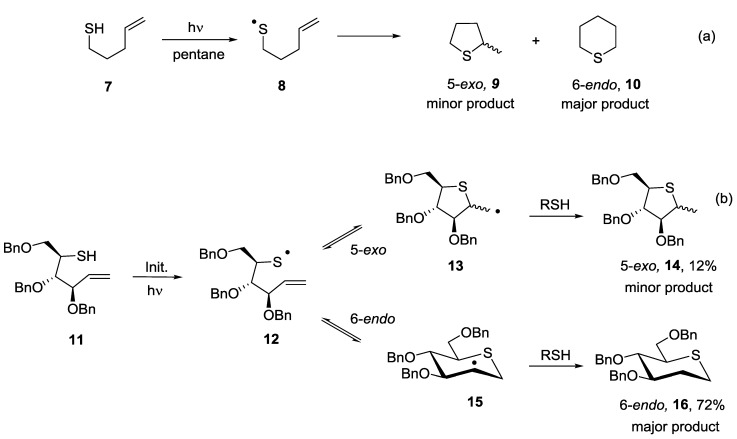
Results for 5-*exo vs*. 6-*endo* cyclisation reaction onto a terminal alkene.

The 5-*exo vs*. 6-*endo* cyclisation reaction is the most widely studied of the intramolecular thiol-ene cyclisation reactions. The first detailed studies into the cyclisation reactions of unsaturated mercaptans involving thiyl radicals were carried out by 1967 by Surzur and co-workers and employed 4-penten-1-thiol **7** as a substrate [21]. The thiyl radical generated from **7** upon UV-light irradiation, cyclized to give a mixture of both five- and six-membered rings, with the six membered *endo* product being formed preferentially, in particular under conditions favouring thermodynamic control (Scheme 3, Equation (a)). Our research group has recently investigated the intramolecular thiol-ene reaction as an efficient strategy for accessing novel synthetic thiosugars as putative glycosidase inhibitors [22,23]. The highly functionalised thiol **11** was prepared in four steps from commercially available *O*-benzyl protected arabinose. The radical mediated cyclisation reaction onto the terminal alkene was carried out under mild activating conditions involving UV irradiation of a solution of **11** in DMF at room temperature in the presence of 10 mol % of 2,2-dimethoxy-2-phenylacetophenone (DPAP) as a radical initiator and 10 mol % of 4-methoxyacetophenone (MAP) as a photosensitizer. Despite the complexity of this system and the potential for competing hydrogen abstraction processes, the optimised cyclisation reaction furnished the 6-*endo* product **16** in 72% isolated yield and the 5-*exo* product **14** in 12% as a mixture of diastereoisomers (Scheme 3 Equation (b)). The results of this cyclisation reaction were in good agreement with the observations of Surzur, in that the 6-*endo* process was favoured. These examples verified for the first time that the intramolecular thiol-ene reaction could be employed for thiosugar synthesis.

**Scheme 4:**
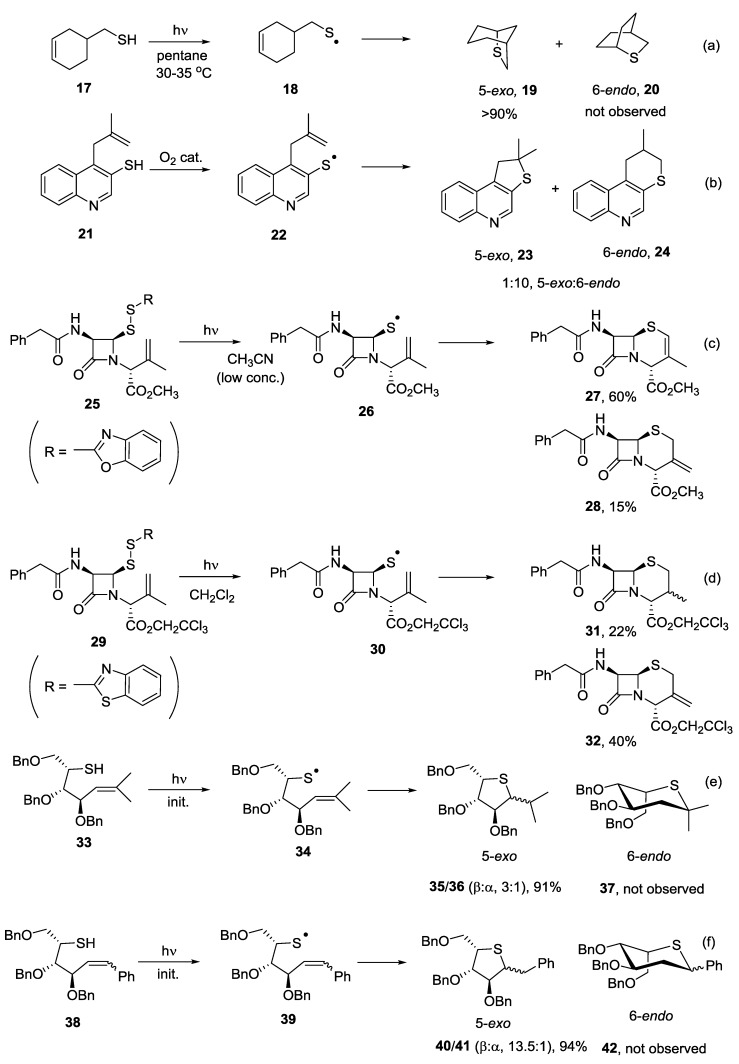

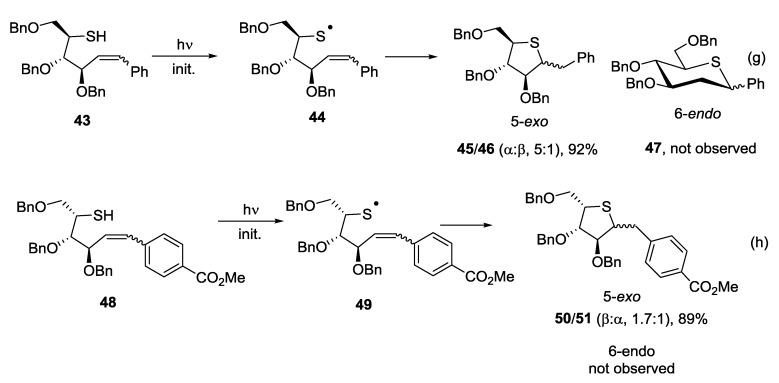
Results for 5-*exo vs*. 6-*endo* cyclisation reaction onto substituted alkenes.

The formation of a mixture of isomers resulting from competing 5-*exo* and 6-*endo* cyclization processes onto unsubstituted terminal alkenes has been attributed to the rapid reversibility of the radical addition step. The use of substituted alkenes can be employed to effectively promote the cyclisation reaction towards a single product. A highly regioselective cyclisation reaction has been reported for the formation of thia-6-bicyclo[3.2.1]octane (**19**, Scheme 4, Equation (a)). In this process, none of the 6-*endo* product **20** was observed [24]. The radical cyclisation reaction of thiol **21**, itself formed upon thermal rearrangement of methallyl-3-quinolylsulphide, furnished the 6-*endo* product **24** as the major product (Scheme 4, Equation (b)) [25,26]. Ionic conditions promoted formation of the 5-*exo* product via a thermal thia-Claisen rearrangement [27,28]. Maki and co-workers reported intramolecular thiol-ene coupling reactions as a powerful strategy for the synthesis of penicillin derivatives. Upon irradiation in acetonitrile, penicillin derivative **25** furnished a mixture of 3-methylenecepham methyl ester **27** and 3-methyl-2-cephem methyl ester **28** (Scheme 4, Equation (c)).

The proposed mechanism involved homolytic cleavage of the disulphide bond upon irradiation, followed by 6-*endo* cyclization of the resulting thiyl radical and subsequent hydrogen atom abstraction to give the two isomers [29]. In a later study, the same authors determined that at high concentration, intermolecular addition of a thiyl radical competes with the photocleavage process and gives rise to a carbon centered radical that undergoes homolytic substitution at the sulfur atom to furnish penams **31** and **32** (Scheme 4, Equation (d)) [30]. The ability to control regeoselectivity via alkene substitution was further developed for the preparation of C-linked thiasugars [22]. Preparation of the isopropylidene olefin **33** was carried out using a Wittig reaction. The free-radical cyclization reaction proceeded in 91% yield with complete regioselectivity and good diastereoselectivity in favor of the 1,2-*trans* product **35** (Scheme 4, Equation (e)). The 5-*exo* product was highly favored in this cyclisation pathway due to the formation of a more stabilised tertiary radical intermediate, the competing 6-*endo* product was not observed. Introduction of a phenyl group onto the alkene also promoted the 5-*exo* cyclisation. For both the D-sugar **43** and the L-sugar **38**, the free-radical cyclization reaction proceeded in high yield with very high diastereoselectivity, again in favor of the 1,2-*trans* products **45** and **40** (Scheme 4, Equations (f) and (g)). Finally a methylbenzoate containing system was prepared and the free radical cyclisation was carried out. Once again, the two diasteriomeric products **50** and **51** resulting from the 5-*exo* cyclisation pathway were the only products observed (Scheme 4, Equation (h)). In general for these systems it can be proposed that the regioselectivity of the cyclisation can be directed through the substitution pattern on the alkene.

**Scheme 5:**
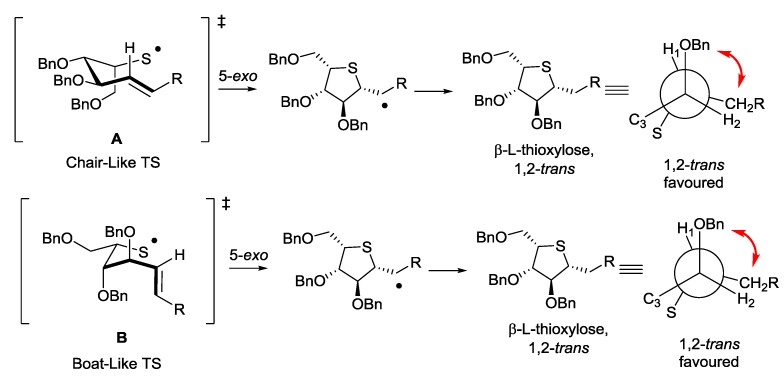
Potential transition state structures for the 5-*exo* thiyl radical cyclisation.

The diastereoselectivity of the free-radical cyclisation reaction may be rationalised in terms of the transition state structures that can be adopted. Beckwith, Houk and others have previously reported that hex-5-enyl cyclizations normally proceed through either a “chair-like” or a “boat-like” transitions state [31,32]. RajanBabu has carried out detailed studies on the cyclization of hex-5-enyl radicals derived from carbohydrates and these studies have concluded that in systems with a C_4_ substituent present, the local allylic conformation will dictate the formation of the “chair-like” or “boat-like” transition states and the conformation that results in the least allylic strain will dominate [33,34,35]. The chair like transition state **A** or the boat-like transition state **B**, both of which give rise to the observed 1,2-trans products, may be considered for these cyclisation reactions (Scheme 5).

**Scheme 6:**
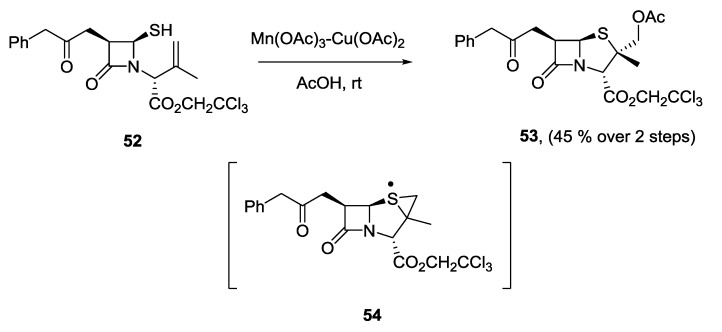
Metal-catalysed cyclisation of penicillin derivatives.

As was outlined in the introduction, thiyl radicals may also be generated on treatment of a thiol with a metal. Cabri and co-workers have reported the transition metal-mediated cyclisation of penicillin derivatives [36]. Initially, the authors reported that both Fe(III) and Mn(III) could promote the cyclisation, they later developed catalytic Fe(III)−Cu(II) and Mn(III)−Cu(II) variants of this reaction, which furnished α-methyl-substituted penicillins in a highly stereoselective manner (Scheme 6) [37,38]. In the work of Maki, the cyclization was proposed to occur through a 6-*endo* process, however Gordon [39] proposed a 5-*exo* cyclization, followed by rearrangement to give **54** as a common intermediate for five- and six-membered ring formation.

### 5.3. 6-exo vs. 7-endo Systems

**Scheme 7:**
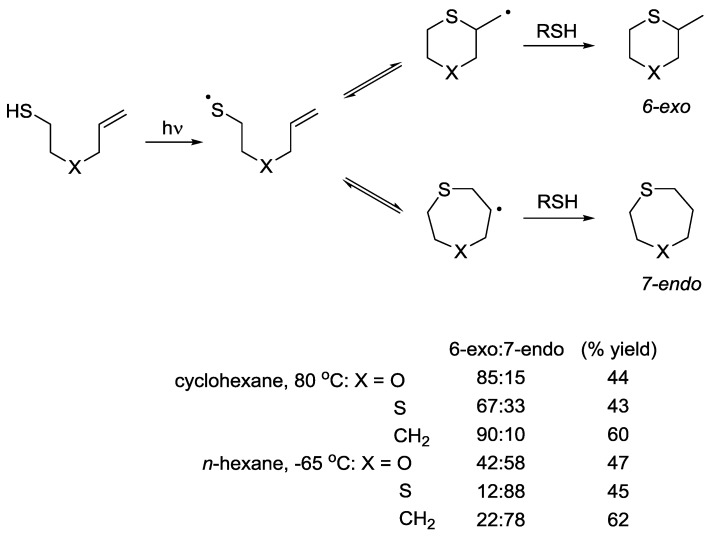
Effect of temperature on regioselectivity of radical cyclisation as determined by Surzur *et al.*

The 6-*exo vs*. 7-*endo* cyclisation pathway was investigated in detail by Surzur and co-workers. Similar to the 5 *vs*. 6 systems described above, significant variation in product distribution could be achieved through variation of the reaction conditions and the alkene. Detailed investigations into systems of the general structure CH_2_=CH–CH_2_–X–(CH_2_)_2_–SH (where X = O, S, CH_2_) revealed a dramatic effect of temperature on the regioselectivity of the cyclisation reaction (Scheme 7) [21,40].

**Scheme 8:**
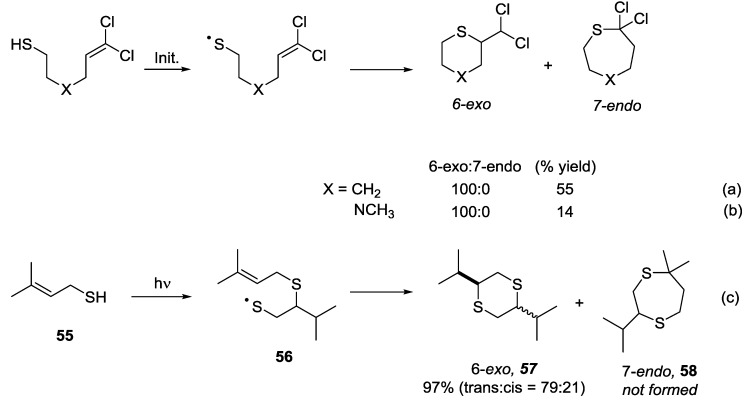
Results for 6-*exo vs*. 7-*endo* cyclisation reaction onto substituted alkenes.

Under high temperature conditions, designed to promote formation of the thermodynamic product, the six-membered rings were consistently formed preferentially over the seven-membered ring systems. At low temperatures, under conditions where kinetic control is dominant, the 7-*endo* products were favoured. When X = N, the dominant product was that resulting from an initial hydrogen abstraction step [41,42]. This represents a reversal of the trends observed for the 5-*exo vs*. 6-*endo* case where the 5-*exo* product was favoured under kinetic conditions and the 6-*endo* product under thermodynamic conditions. These results highlight the challenges in making general statements regarding the predicted outcome of intramolecular thiol-ene reactions involving addition reactions onto unsubstituted terminal alkenes. In a trend similar to that observed for the 5-*exo vs*. 6-*endo* systems, very selective addition reactions in favour of the *exo* products have been observed in systems where the alkene is substituted. Formation of the *endo* product is completely suppressed when X = CH_2_ or NCH_3_ in the chlorinated substrates [43,44]. This is presumably due to the stabilising effect of the chlorine atoms on the intermediate alkyl radical species (Scheme 8, Equations (a) and (b)). Tanaka and co-workers have reported that photochemical activation of a solution of prenyl mercaptan in hexane results in almost quantitative conversion to dithiane **57**. The prenyl undergoes an initial dimerization step via intermolecular thiol-ene addition and this is followed by an intramolecular cyclisation that furnishes the 6-*exo* product exclusively (Scheme 8 Equation (c)) [45]. Unusually for this type of system, no polymerisation products were observed. The selectivity towards the 6-*exo* product may be attributed to the stabilising effect of the two methyl groups on the intermediate radical. This is analogous to the carbohydrate derived system where the 5-*exo* process was promoted using a similar approach (Scheme 4, Equation (e)).

### 5.4. 7-exo vs. 8-endo Systems

**Scheme 9:**
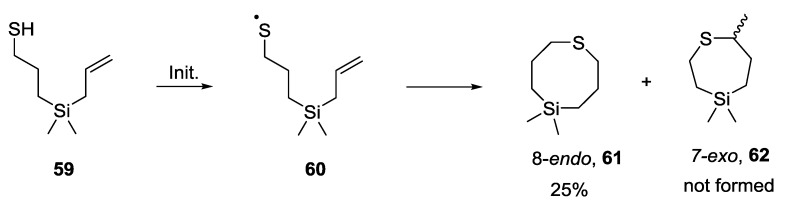
Results for 7-*exo vs*. 8-*endo* cyclisation reaction.

Cyclisation reactions to larger ring sizes have also been investigated. Weber and co-workers have reported an 8-*endo* cyclisation reaction of a thiyl radical onto a terminal alkene [46,47]. The thiyl radical was generated upon irradiation of hydrogen sulphide and dimethyldiallylsilane in pentane at −78 °C. The 8-*endo* product **61** was observed exclusively in 25% yield, none of the 7-*exo* product **62** was isolated (Scheme 9). It can be assumed that at −78 °C the reaction is essentially under kinetic control and the results are consistent with those observed in the 5 *vs*. 6 systems; however the low yield makes it difficult to draw strong conclusions from this data. It is also unknown if the presence of the bulky silicon atom in the ring has any effect on the regioselectivity of the cyclisation reaction, however it is likely that the C-Si bond length (1.87 Å), would favour formation of the larger ring.

### 5.5. Macrocycles

**Scheme 10:**
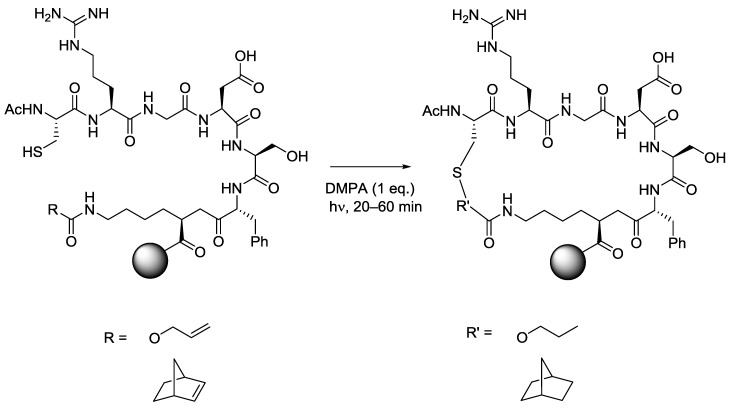
Example of intramolecular thiol-ene reaction applied to cyclic peptide synthesis.

Based on the efficiency of the intermolecular thiol-ene reaction for polymerisation and ligation reactions, it is not surprising that the methodology has been employed to prepare thioether linked macrocycles. While formally an intramolecular cyclisation process, the kinetics and thermodynamics of the addition reaction are more closely related to the intermolecular process and cannot be directly compared to the intramolecular cyclisations of smaller rings. Anseth and co-workers have reported on-resin cyclization of peptides using intramolecular thiol-ene photochemistry [48,49]. Cysteine amino acid residues were exploited as the source of the thiyl radical, peptide bound terminal alkene and norbornene derivatives were used for the site of addition. The cyclised peptide products were isolated in yields of 37% and 24% respectively (Scheme 10).

## 6. Conclusions and Future Directions

Since the earliest reports of the intramolecular thiol-ene reaction, the reaction has been studied in detail for a number of varying chain lengths and diverse substrates. It is clear from these results that the methodology offers an extremely mild and efficient strategy for accessing sulphur containing heterocycles of varying ring sizes in the presence of a wide range of functional and protecting groups. Improvements in radical initiators and an enhanced understanding of the reaction kinetics and thermodynamics have facilitated the application of the intramolecular thiol-ene to complex systems including large peptides. While the outcome of cyclisation reactions onto unsubstituted terminal alkenes remains difficult to predict, substitution of the alkene group allows for control over regioselectivity for most ring sizes. In addition to high regioselectivity, a high degree of stereoselectivity can also be achieved through careful design of the starting material and control of the reaction conditions. The ability of these reactions to be carried out in an aqueous environment should give rise to “green” strategies for heterocycle synthesis, reducing the use of protecting groups and organic solvents. Cascade cyclisation reactions involving intramolecular thiyl radical reaction steps have yet to be exploited. The use of thiyl radicals in intramolecular cyclisation pathways offers numerous possibilities for the construction of complex molecular architecture.
